# The Circadian Syndrome: is the Metabolic Syndrome and much more!

**DOI:** 10.1111/joim.12924

**Published:** 2019-06-10

**Authors:** P. Zimmet, K. G. M. M. Alberti, N. Stern, C. Bilu, A. El‐Osta, H. Einat, N. Kronfeld‐Schor

**Affiliations:** ^1^ Department of Diabetes Central Clinical School Monash University Melbourne Vic. Australia; ^2^ Sagol Center for Epigenetics and Metabolism Tel Aviv Medical Center Tel Aviv Israel; ^3^ Imperial College London UK; ^4^ School of Zoology Tel Aviv University Tel Aviv Israel; ^5^ Department of Pathology The University of Melbourne Parkville Vic. Australia; ^6^ Hong Kong Institute of Diabetes and Obesity Prince of Wales Hospital The Chinese University of Hong Kong Hong Kong SAR China; ^7^ School of Behavioral Sciences Tel Aviv‐Yaffo Academic College Tel Aviv Israel

**Keywords:** circadian clock, diabetes, metabolic syndrome

## Abstract

The Metabolic Syndrome is a cluster of cardio‐metabolic risk factors and comorbidities conveying high risk of both cardiovascular disease and type 2 diabetes. It is responsible for huge socio‐economic costs with its resulting morbidity and mortality in most countries. The underlying aetiology of this clustering has been the subject of much debate. More recently, significant interest has focussed on the involvement of the circadian system, a major regulator of almost every aspect of human health and metabolism. The Circadian Syndrome has now been implicated in several chronic diseases including type 2 diabetes and cardiovascular disease. There is now increasing evidence connecting disturbances in circadian rhythm with not only the key components of the Metabolic Syndrome but also its main comorbidities including sleep disturbances, depression, steatohepatitis and cognitive dysfunction. Based on this, we now propose that circadian disruption may be an important underlying aetiological factor for the Metabolic Syndrome and we suggest that it be renamed the ‘Circadian Syndrome’. With the increased recognition of the ‘Circadian Syndrome’, circadian medicine, through the timing of exercise, light exposure, food consumption, dispensing of medications and sleep, is likely to play a much greater role in the maintenance of both individual and population health in the future.

## Graphical abstract

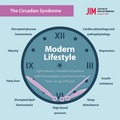

## Introduction

### The circadian clock and metabolic derangement

The circadian system is the major regulator of almost every aspect of human health and metabolism. The human brain has a master ‘Body Clock’ which resides in the suprachiasmatic nucleus (SCN) of the hypothalamus and it determines our daily rhythms, a phenomenon also widely described in nature in almost all living organisms 1, 2. This master clock regulates the body's metabolism through controlling body functions, and synchronizing peripheral clocks in almost all cells in the body, including the body's key tissues such as the heart, liver, muscle and adipose tissue 3, 4, 5, 6.

Circadian rhythms are affected by environmental signals. Light is the main cue influencing the SCN master circadian clock, turning on or off genes that control the individual's internal clock function. Other environmental factors include temperature change and nutritional intake, which mainly affect peripheral clocks 4, 7.

An important background perspective to this scenario is the escalating global epidemics of type 2 diabetes mellitus (T2DM) 8 and cardiovascular disease (CVD) 9 against the dramatic changes that have occurred in Western and even traditional‐living societies over recent decades with globalization and modernization 10. This includes changes in light exposure resulting from the extensive use of artificial light (‘light pollution’) 11, 12, 13, controlled ambient temperature and constant food availability 14, societal and workplace stresses, increasing shift work in the workplace and industry, jet travel with time zone changes and changes in nutrition. In the light of this, it has been suggested that the resulting circadian rhythm disturbances may be a major contributor to the contemporary global epidemics of T2DM, CVD and obesity 3, 5, 6, 7, 11, 15, 16, 17, 18, 19.

The circadian system is one of the major regulators of human health and metabolism 3, 4, 20. It regulates gene expression, release of various hormones, body temperature, activity pattern, energy expenditure and other important body functions. This being the case, it is not surprising that there has been significant interest in the relationship of circadian disruption with glucose metabolism 5, 13, 15, 16, 18, 21, 22 and other components of the Metabolic Syndrome 6, 17, 19, 22, 23.

#### Metabolic syndrome components and circadian disruption

The Metabolic Syndrome, the cluster of cardio‐metabolic risk factors and comorbidities, is responsible for large health and socio‐economic costs in most nations mainly for the resulting morbidity and mortality from noncommunicable diseases (NCDs) including obesity, T2DM, CVD, cancer and mood disorders 8. It can create a ‘perfect storm’ that is likely to cripple health budgets in many nations. This cardio‐metabolic risk cluster is commonly associated with comorbidities including sleep disturbances and depression 24, 25, 26, cognitive disorders 27, 28 and nonalcoholic fatty liver disease (NAFLD) 29. Abnormal circadian rhythms have been associated with obesity, T2DM, CVD and hypertension 3, 23, 30, all components of the Metabolic Syndrome. Shift workers or people who sleep poorly are more likely to develop obesity and T2DM due to circadian clock disruption 31, 32, 33, 34, 35, 36, 37.

There is continuing debate and dispute as to whether there is a common underlying aetiology that could explain this clustering of cardio‐metabolic risk determinants, and indeed the associated comorbidities. Suggestions on aetiology have included insulin resistance 38, a central obesity driven proinflammatory state 39 and genetics 40 but little consensus on aetiology exists. Noteworthy are the earlier suggestions that disturbances in circadian rhythm might play a role 23, 41, 42, and we are now proposing the concept of the Circadian Syndrome as the Metabolic Syndrome and more.

In his 2011 paper ‘The Metabolic Syndrome: time to get off the merry‐go‐round’ 43, Gerry Reaven agreed with our criticism of the syndrome for the lack of consistency of the components of the syndrome, their diagnostic criteria/cut‐off points and the number of components needed to make a diagnosis as suggested by different organizations and researchers 39, 44, 45. Despite this consensus, he questioned the value of the Metabolic Syndrome as an effective way to identify apparently healthy individuals at an increased risk to develop CVD and T2DM. In addition, he did not move away from the clustering of the different components occurring more than by chance alone. He argued that little of the vast published information pertaining to the Metabolic Syndrome had provided new pathophysiological insight, nor supported the clinical utility of this syndrome as a diagnostic category. In making these points, Reaven actually strengthened our argument for the establishment of the Circadian Syndrome, which we discuss below.

The Metabolic Syndrome only recognizes a few of the aetiological components of what constitutes those of our proposed Circadian Syndrome and it says virtually nothing about its actual aetiology. By including the comorbidities as new components and bringing in the role of the circadian system, we now move towards a much stronger aetiological basis. In addition, it provides a more logical pathophysiological construct and a clinical platform for intervention and prevention of a variety of noncommunicable diseases, and not just CVD and T2DM.

The late Gerry Reaven made landmark contributions in the better understanding of the role of insulin resistance in the aetiology of the cardio‐metabolic cluster. In his 2011 contribution to the Journal of Internal Medicine 43, he asked if there was any reason why the Metabolic Syndrome should not be given its well‐deserved send‐off?

With this suggestion, we can agree as we believe the Circadian Syndrome provides a much more rational substitute for the controversy that has surrounded the Metabolic Syndrome for several decades. And by this action, it has an important rationale for intervention and prevention through a better understanding of the role of circadian dysrhythmia.

More recently, epigenetics has been suggested to be involved as the driver of the cardio‐metabolic cluster 46, 47, 48. DNA methylation provides a mechanism by which environmental factors like diet and exercise can modify genetic predisposition to disease. Reviewing DNA methylation in metabolic disorders, Barries and Zierath noted that it is a major epigenetic modification that controls gene expression in physiologic and pathologic states cluster 47. They pointed out that metabolic disorders such as diabetes and obesity are associated with profound alterations in gene expression through DNA methylation caused by genetic and environmental factors.

Epigenetic changes such a DNA methylation and histone modification may be transmitted across generations either directly by persisting through meiosis or indirectly through replication in the next generation of the environmental conditions in which the epigenetic change occurred 46, 49. Furthermore, Gluckman and his colleagues point out that the current environment increases the risk of chronic metabolic diseases and CVD 39. This underlines that epigenetic processes are a key mechanism that alter the individual's susceptibility to the development of NCDs like T2DM and CVD 46, 50. It is noteworthy that these epigenetic modifications can be transmitted intergenerationally. For example, studies in *Psammomys obesus* have shown parental diet regulates DNA and RNA methylation and the expression of genes implicated with the increased risk of obesity in offspring 51, 52.

The potential role of circadian dysrhythmia and epigenetics will be discussed in more detail later.

#### The Metabolic Syndrome: the controversy on its relevance and definition

The term Metabolic Syndrome remains the most widely accepted name to describe this cluster of metabolically related CVD risk factors 40 despite vigorous attempts to discard the syndrome as a clinical entity 43, 53. In 2005, a joint statement of the American Diabetes Association (ADA) and the European Association for the Study of Diabetes (EASD) ‘*The Metabolic Syndrome: time for a critical appraisal*’ 54 raised several questions regarding the status of the Metabolic Syndrome, further driving the debate as to whether it existed as an entity in its own right. The conclusion was that: ‘……. *while there was no question that certain CVD risk factors are prone to cluster, they found that the Metabolic Syndrome had been imprecisely defined, there is a lack of certainty regarding its pathogenesis, and there is considerable doubt regarding its value as a CVD risk marker. Our analysis indicates that too much critically important information is missing to warrant its designation as a syndrome*’ 54.

Quite independent of this, several separate and conflicting components and diagnostic criteria for the Metabolic Syndrome had been proposed by different organizations and individuals over past decades. This created a lot of confusion, affecting adversely attempts to obtain universal consensus on the aetiopathogenesis, the key components of the syndrome for its definition and diagnostic criteria, and the expected longer‐term outcomes 43, 44.

In 2009, to clarify issues regarding the agreed essential components and their diagnostic criteria, a joint statement, ‘Harmonizing the Metabolic Syndrome’ 45 was issued by a consortium comprising the International Diabetes Federation (IDF) Task Force on Epidemiology and Prevention, the National Heart, Lung, and Blood Institute, the American Heart Association, the World Heart Federation, the International Atherosclerosis Society; and International Association for the Study of Obesity.

The objective was to provide the basis for confirming the key components that defined the Metabolic Syndrome (Table 1). The authors concluded that*…. ‘A cluster of risk factors for cardiovascular disease and type 2 diabetes mellitus, which occur together more often than by chance alone, have become known as the Metabolic Syndrome. The risk factors include raised blood pressure, dyslipidaemia (raised triglycerides and lowered high‐density lipoprotein cholesterol), raised fasting glucose, and central obesity’*. Furthermore, they stated that: *‘A single set of cut points would be used for all components except waist circumference, for which further work is required. In the interim, national or regional cut points for waist circumference can be used*’ 45.

**Table 1:** The ‘harmonized’ Metabolic Syndrome: criteria for clinical diagnosis^a^

Measure	Categorical cut points
Elevated waist circumference^b^	Population ‐ and country‐specific definitions
Elevated triglycerides (drug treatment for elevated triglycerides is an alternate indicator	≥150 mg dL^−1^ (1.7 mmol L^−1^)
Reduced HDL‐C (drug treatment for reduced HDL‐C is an alternate indicator)	<40 mg dL^−1^ (1.0 mmol L^−1^) in males; <50 mg dL^−1^ (1.3 mmol L^−1^) in females
Elevated blood pressure (antihypertensive drug treatment in a patient with a history of hypertension is an alternate indicator)	Systolic ≥ 130 and/or diastolic ≥ 85 mm Hg
Elevated fasting glucose^c^ (drug treatment of elevated glucose is an alternate indicator)	≥100 mg dL^−1^ (5.6 mmol L^−1^)

a Adapted from reference 49.

b It is recommended that the IDF cut points be used for non‐Europeans and either the IDF or AHA/NHLBI cut points used for people of European origin until more data are available.

c Most patients with type 2 diabetes mellitus will have the Metabolic Syndrome by the proposed criteria.

Nevertheless, an important issue still remained as to the uncertainty whether there is a common and central aetiological feature to explain the clustering of these risk factors. Not only this but also the accompanying commonly associated comorbidities of sleep disturbance, depression and NAFLD. Whilst numerous suggestions have been put forward as discussed earlier, none have provided an acceptable explanation.

**Figure 1:**
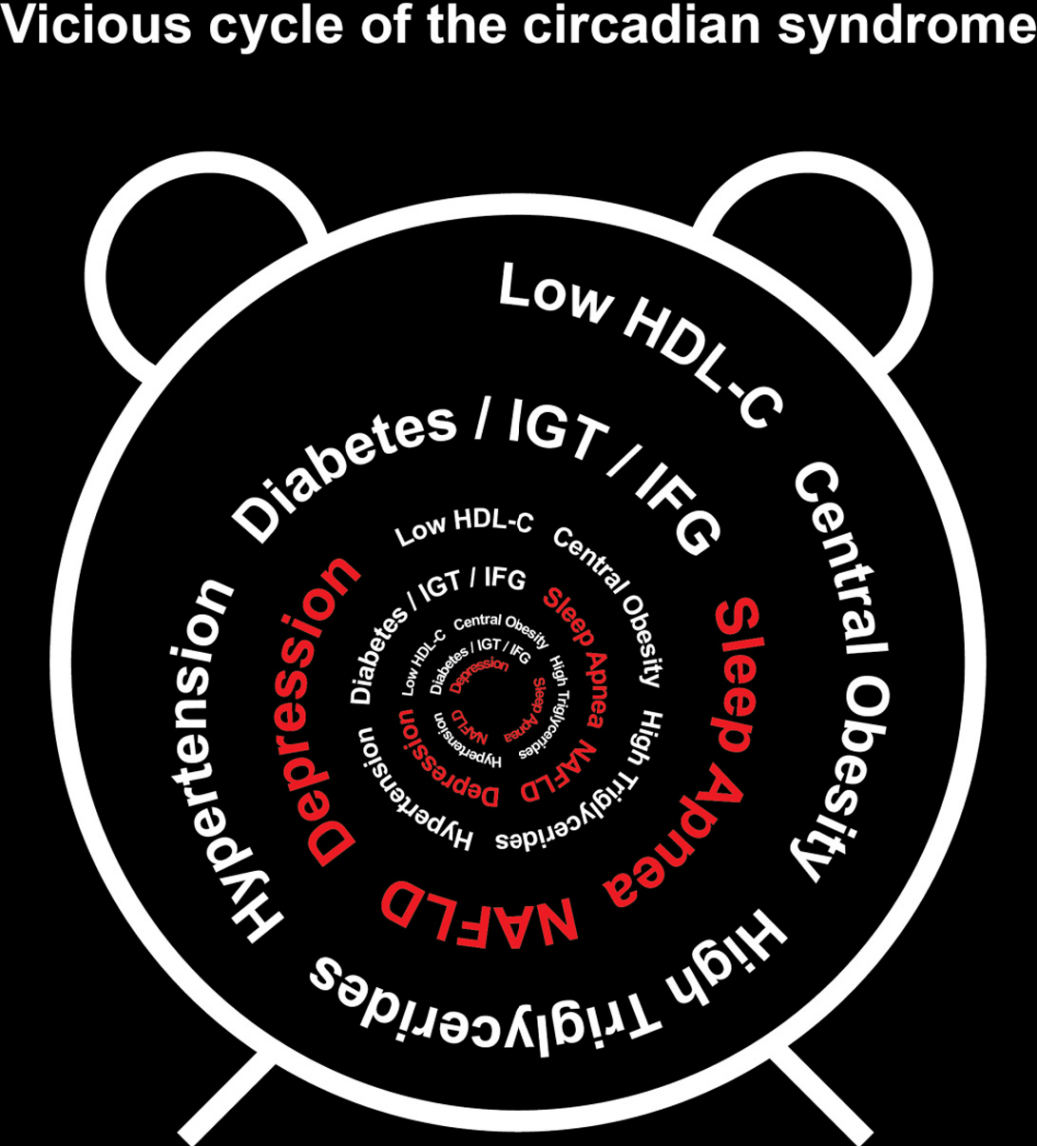
Components of the proposed Circadian Syndrome.

This provides the basis for our proposal discussed below relating on the evidence that links not only the cardio‐metabolic cluster but also its associated comorbidities. Increasing research links them with disturbances of circadian rhythm and epigenetics. Therefore, we now propose that combined, that is, the Metabolic Syndrome cluster and the comorbidities should be recognized together and designated as the ‘Circadian Syndrome’ (Fig. 1).

There is mounting evidence to link these cardio‐metabolic risk factors and the comorbidities with circadian rhythm disturbances which suggests all, or the majority, of the cluster components could have a common aetiology. It suggests that disturbances of the body's circadian rhythm can play a central role. Thus, it is reasonable to propose circadian disruption may drive this frequently seen cluster of risk factors and disorders that include T2DM, CVD, ageing, hypertension, sleep apnoea, NAFLD and depression as well as some other CVD risk factors including dyslipidaemia 3, 17, 18, 19, 23.

Because of this and increasing evidence of linkage to the body's circadian rhythm, we are proposing that the existing ‘harmonized’ version of the Metabolic Syndrome (Table 1) be modified by including the associated comorbidities and it be renamed the ‘Circadian Syndrome’ and the associated comorbidities be included (Fig. 1).

What is the justification for this proposal? The review below provides the basis for our suggestion that disturbances in circadian rhythm may be the basis for the clustering of what might on the surface appear to be separate biological phenomena. Recent developments in epigenetics may also provide the basis for a better understanding of the pathophysiological mechanisms underlying what to date we have called the Metabolic Syndrome 4, 47.

Interest in this concept of the critical role of circadian rhythm in human health and disease is not new. In 2006, Staels, in his paper ‘*When the clock stops ticking, Metabolic Syndrome explodes’*, drew attention to studies, recent at that time, showing altering circadian rhythmicity also resulted in pathophysiological changes resembling the Metabolic Syndrome 23. In 2008 in an editorial, ‘*The clock stopped, never to go again………’* Grant suggested that a full understanding of the endogenous body clock's role might have major implications regarding ‘our view of complex body disorder such as diabetes and cardiovascular disease’^41^. This statement was prophetic given that circadian rhythms are so closely entwined with the sleep–wake cycle, feeding behaviour, body temperature and a variety of endocrine functions and key organs including the heart, kidney and liver. Clearly then, disruption of the central body clock and consequently the peripheral clocks with the resulting metabolic disruptions could have important implications for human health.

Mentioned earlier was the potential association between the escalating global epidemics of CVD and T2DM and the dramatic changes that have occurred in societies over recent decades. We see this scenario as pivotal in the pathogenesis of obesity, T2DM and CVD and in the light of the modern epidemics of NCDs 8, 55. Additionally, despite the long‐standing focus on the role of gene–environment influences in NCD aetiology, a more contemporary view is now the importance of epigenetics 4, 8, 20, 46, 47. For example, DNA methylation, an important determinant of epigenetic change regulates tissue‐specific expression of genes described recently in adipose tissue after one night of sleep loss 56. The implications of these findings suggest new regulatory mechanisms involved in chronic sleep loss may promote weight gain.

### Circadian disruption and associations with risk factors

Herein we discuss research findings relating to the proposed Circadian Syndrome risk components and comorbidities (Table 2).

**Table 2:** Circadian system involvement in key cardio‐metabolic risk factors and outcome

	Human	Sand rats
Cardiovascular‐related components	Arrhythmia	
	Onset of myocardial infractions	
	Sudden cardiac death 19, 61	
Hypertension	Lack of nocturnal decline in blood pressure 30, 62	High blood pressure 22
Lipids	Disrupted lipid homeostasis 69, 70	
Obesity	Disrupted diurnal rhythms in the white adipose tissue transcriptome 73	Obesity 22
Blood glucose levels	Disrupted daily rhythms in blood glucose levels and glucose intolerance 17, 21, 75	Disrupted daily rhythms in blood glucose levels and glucose intolerance 22
Fatty liver	Disrupted triglyceride accumulation, inflammation, oxidative stress and mitochondrial dysfunction 29	
Depression	Circadian disruption increases the incidences of depression 86, 87	Circadian disruption increases the incidences of depression 22, 83, 84, 105, 106, 107, 108, 109, 110
Sleep disturbances	Circadian disturbances result in sleep loss and mistimed sleep 56, 97	

#### Cardiovascular‐related components

Circadian oscillations have been reported for both physiological CVD regulation including heart rate and blood pressure 57, for cardiac disorders including arrythmias 58, 59, and there is circadian variation in frequency of onset of myocardial infarction and sudden cardiac death 19, 60.

Human and animal studies suggest that circadian clocks are not only important drivers of rhythms in CVD physiology and pathology but also for disease prevention and management 19, 61. It has been suggested that that clock genes likely influence cardiac physiology and pathophysiology 19, 61. The basis being the close association between obesity, diabetes mellitus and CVD as key components of the Metabolic Syndrome, as well as the direct effects that the circadian clock imposes on myocardial function. The effect is direct through the cardiomyocyte circadian clock and indirect through central and peripheral clock effects on behaviour and the neurohumoral environment.

#### Hypertension

In most healthy subjects, there is diurnal variation in blood pressure with systolic and diastolic blood pressures which parallels, in general, the circadian variation of blood pressure regulating systems such as sympathetic activity, cortisol and aldosterone 62. Blood pressure dips at night, typically at the end of the night, and on arousal, blood pressure surges 30. Lack of nocturnal decline in blood pressure, in the form of nocturnal hypertension or ‘nondipping’, carries a significant risk of cardiovascular morbidity and mortality which largely exceeds that of office‐based hypertension 30, 63. Nondipping hypertension has been associated with insulin resistance, obesity, the Metabolic Syndrome and type 2 diabetes 64. Sleep apnoea may be one important common denominator linking obesity and the Metabolic Syndrome to nondipping hypertension. Further, better oxygenation through night‐time continuous positive air pressure reduces nocturnal hypertension 65 and reduces catecholamines and aldosterone secretion 66. Timing of treatment to body rhythms is a means of individualizing and optimizing the treatment of hypertension and may a constitute an effective option to reduce CVD risk 67. Furthermore, variation in the day–night blood pressure pattern appears to be an important predictor of both target organ damage and cardiovascular events 68.

#### Lipids

Lipids have a critical role in cardiovascular and metabolic disorder risk and management including their role as components of the Metabolic Syndrome. As a result, mounting evidence now suggests that circadian clocks play a major role in lipid homeostasis both in humans and animals, particularly in relation to CVD but also nutrition and other body functions. Detailed reviews of this subject are available elsewhere 69, 70.

#### Obesity

Apart from the central hypothalamic clock, there are peripheral clocks in key tissues including adipose tissue. Given the close linkage of obesity, especially central (abdominal) as a key component of the Metabolic Syndrome, obesity plays an important role in risk of T2DM and insulin sensitivity as well as CVD 39. A role for circadian rhythms, including the circadian clock within the adipocytes, in the development of obesity was described 17, 71, 72, 73. As noted earlier, there is also a close relationship with obesity in terms of risk for obstructive sleep apnoea, a key comorbidity.

#### Blood glucose levels, glucose tolerance and circadian effect

That glucose tolerance has a diurnal pattern has been known for many years. In 1974, we reported on diurnal variation in oral glucose tolerance, plasma insulin and nonesterified fatty acid levels 74. Blood glucose levels were higher in the afternoon indicating that glucose tolerance is impaired during the evening hours because of the body's circadian rhythm. Plasma glucose concentration results from the balance between glucose input (from food or hepatic glucose production) and its uptake by muscle and adipose tissue. The concentration is controlled by the SCN both indirectly, via controlling feeding rhythms (which effect glucose and insulin rhythms), and directly, by affecting glucose production and utilization and insulin sensitivity in tissues like liver, muscle and pancreas 17, 21, 75. This rhythm is independent of food availability and depends on a functioning central clock 21, 76, 77. This indicates that disrupted circadian rhythms will disrupt glucose homeostasis, and that diabetes testing with the oral glucose tolerance test, and indeed the fasting glucose, should always be performed in the morning otherwise a false diagnosis of diabetes may be the outcome 74.

### Comorbidities and the Circadian Syndrome

#### Fatty liver

Circadian disruption may play a role in the pathogenesis of nonalcoholic fatty liver disease, which is now the commonest chronic liver disease in Western nations 78. It is strongly associated with the Metabolic Syndrome and its prevalence continues to rise along with that of the obesity and Metabolic Syndrome epidemics. Metabolic homeostasis is linked to the circadian clock with the clock involved in regulation of hepatic triglyceride accumulation, inflammation, oxidative stress and mitochondrial dysfunction 29, all of which may contribute to the pathogenesis of NAFLD.

#### Depression

Depression is a well‐established comorbidity associated with T2DM 79, 80, 81, 82. Nearly all patients with affective disorders including depression show significant disruptions in circadian rhythms 83, 84, 85, 86, 87. Depression is related to disturbances of the circadian clock, and light therapy and other treatment affecting the circadian clock are used as a therapy for depression 83, 88, 89, 90, 91, 92, 93, 94, 95, 96. It is likely that these treatments allow resynchronization of circadian rhythm.

#### Sleep disturbances

Risk of obesity, T2DM and Metabolic Syndrome is elevated in people who suffer from chronic sleep loss or are shift workers. Almoosawi and her colleagues suggest these situations result in misalignment between the sleep–awake, fasting–feeding cycles, and the light–dark cycle 97. This results in physiologic processes affected by circadian imbalance including glucose, lipid metabolism and blood pressure. These manifests then heightened risk of developing T2DM and CVD.

A recent study by Cedernaes *et al*. 56 demonstrated epigenetic changes in DNA methylation in the clock genes. This only occurs in conditions like obesity and T2DM. Whilst DNA methylation modifications are believed to confer ‘metabolic memory’ other studies indicate histone modifications regulate the expression of genes implicated in diabetic complications 98, 99. The findings may contribute to a better understanding of how sleep deprivation and the associated circadian disruption may contribute to the risk of developing obesity.

## Conclusion

The accumulating evidence linking circadian rhythm disruption to lifestyle changes in our society calls for a greater emphasis on this relationship in NCD prevention. Whilst until now the Metabolic Syndrome has been the focus until now, we should not ignore that its comorbidities such as have been discussed earlier are potentially linked as well through disruption of the circadian system and/or epigenetic modifications.

It has long been a challenge for researchers to establish whether the Metabolic Syndrome components and comorbidities have a common aetiological origin. Yet to date, no explanation has revealed a potential mechanism that could explain the possible factor/s. Taking a holistic overview, it seems more than a coincidence that each of the cardio‐risk components and the key comorbidities can be directly linked to circadian disruption involving disturbances of the central and peripheral body clocks. Whilst it is not necessary to have all the cluster components or comorbidities present in an individual, it can enhance both clinical recognition, indeed management and awareness of risk of future CVD and metabolic disease. In addition, recognizing the link with circadian disruption provides the opportunity to dig deeper into understanding the aetiopathogenic pathways leading to what we now suggest as the ‘Circadian Syndrome’. This connection to lifestyle behavioural risk factors has important implications for management of NCDs and prevention.

The Metabolic Syndrome is responsible for huge socio‐economic costs in most countries but often it is not addressed more aggressively despite the health and social costs of the impact of its comorbidities to individuals and society. Therefore, presented in the perspective of the ‘Circadian Syndrome’ and relationship to our present modern way of living may help provide a greater focus on evidence‐based prevention of the global burden of NCDs. This involves a concerted and global attack, particularly on ‘diabesity’ (the twinning of type 2 diabetes and obesity) and CVD, arguably the most important public health issues of our times.

Recognizing the link between lifestyle behavioural risk factors and circadian disruption, risk and aetiology of key NCDs including T2DM and CVD, has important implications for nonpharmacological prevention and therapeutic strategies. With the recognition of the ‘Circadian Syndrome’, circadian medicine through the timing of light exposure, exercise, food consumption, dispensing of medications and sleep, is likely to play a much greater role in the maintenance of both individual and population health in the future (Box 1).

Box 1 **Using a diurnal animal model for studying the circadian syndrome**

Although the Metabolic Syndrome, including type 2 diabetes mellitus (T2DM) and depression, is associated with disturbances of circadian rhythms, most studies of these diseases use nocturnal mice and rats whilst modelling diurnal humans. A diurnal, well‐accepted and extensively studied model for T2DM 48, 51, 100, 101, 102, 103 is the sand rat, (*Psammomys obesus*). When the sand rats are fed normal rodents chow they develop T2DM characterized by enhanced insulin secretion and insulin resistance at the early stages and insulin deficiency at an advanced stage 100, 102. We recently showed that sand rats held outdoors in laboratory cages (where they are exposed to natural environmental conditions) and fed standard rodent chow have robust daily rhythms and do not develop T2DM or depressive‐like behaviour, in contrast to animals held indoors (where the only cycling environmental condition is light) fed the same diet, which show low amplitude rhythmicity and depressive‐like behaviour, and develop T2DM 22. Laboratory conditions are similar in many ways to the conditions we live in modern societies, where we control our ambient temperature and light exposure, where food is available *ad libitum* and where we have no connection to rhythms of other species – food, competitors or predators 104. These unnatural environments, and especially unnatural patterns of light exposure during both day (low light levels indoors) and night (high light levels) directly affect our circadian biology and health, including the development of T2DM and depression. We suggest that using diurnal model animals is advantageous when aiming to study these processes 22, 83.

## Conflict of interest statement

The authors have no conflict of interests.

## References

[1] Kuhlman SJ , Craig LM , Duffy JF . Introduction to chronobiology. Cold Spring Harb Perspect Biol 2018; 10: a033613. 29038118 10.1101/cshperspect.a033613 PMC6120700

[2] Top D , Young MW . Coordination between differentially regulated circadian clocks generates rhythmic behavior. Cold Spring Harb Perspect Biol 2018; 10: a033589. 28893860 10.1101/cshperspect.a033589 PMC6028074

[3] Panda S . The arrival of circadian medicine. Nat Rev Endocrinol 2019; 1. 10.1038/s41574-018-0142-x 30602736

[4] Orozco‐Solis R , Sassone‐Corsi P . Epigenetic control and the circadian clock: linking metabolism to neuronal responses. Neuroscience 2014; 264: 76–87. 24486964 10.1016/j.neuroscience.2014.01.043 PMC6501790

[5] Kalsbeek A , Scheer F. A.. , Perreau-lenz S. , *et al* Circadian disruption and SCN control of energy metabolism. FEBS Lett 2011; 585: 1412–26. 21414317 10.1016/j.febslet.2011.03.021 PMC3095769

[6] Ruger M , Scheer F . Effects of circadian disruption on the cardiometabolic system. Rev Endocr Metab Disord 2009; 10: 245–60. 19784781 10.1007/s11154-009-9122-8 PMC3026852

[7] Li M‐D , Li C‐M , Wang Z . The role of circadian clocks in metabolic disease. Yale J Biol Med 2012; 85: 387. 23012586 PMC3447202

[8] Zimmet PZ , Magliano DJ , Herman WH , Shaw JE . Diabetes: a 21st century challenge. Lancet Diabetes Endocrinol 2014; 2: 56–64. 24622669 10.1016/S2213-8587(13)70112-8

[9] Cleary C *et al* Antidepressive‐like effects of rapamycin in animal models: implications for mTOR inhibition as a new target for treatment of affective disorders. Brain Res Bull 2008; 76: 469–73. 18534253 10.1016/j.brainresbull.2008.03.005

[10] Diamond J . The double puzzle of diabetes. Nature 2003; 423: 599. 12789325 10.1038/423599a

[11] Dominoni DM , Borniger JC , Nelson RJ . Light at night, clocks and health: from humans to wild organisms. Biol Let 2016; 12 10.1098/rsbl.2016.0015 PMC4780560 26888917

[12] Ouyang JQ , Davies S , Dominoni D . Hormonally mediated effects of artificial light at night on behavior and fitness: linking endocrine mechanisms with function. J Exp Biol 2018 10.1242/jeb.156893 PMC5897701 29545373

[13] Versteeg RI *et al* Nutrition in the spotlight: metabolic effects of environmental light. Proc Nutr Soc 2016; 75: 451–63. 27499509 10.1017/S0029665116000707

[14] Stevenson TJ *et al* Disrupted seasonal biology impacts health, food security and ecosystems. Proc Biol Sci 2015 10.1098/rspb.2015.1453 PMC4633868 26468242

[15] Qian J , Caputo R , Morris CJ , Wang W , Scheer FA . Circadian misaligment increases the desire for food intake in chronic shif workers. Sleep 2018; 41: A17.

[16] Qian JY , Scheer F . Circadian system and glucose metabolism: implications for physiology and disease. Trends Endocrinol Metab 2016; 27: 282–93. 27079518 10.1016/j.tem.2016.03.005 PMC4842150

[17] Stenvers DJ , Scheer FAJL , Schrauwen P , la Fleur SE , Kalsbeek A . Circadian clocks and insulin resistance. Nat Rev Endocrinol 2019; 15: 75–89. 30531917 10.1038/s41574-018-0122-1

[18] Noordam R *et al* Associations of outdoor temperature, bright sunlight and cardiometabolic traits in two European population‐based cohorts. J Clin Endocrinol Metab 2019 10.1210/jc.2018-02532 PMC6543772 30759251

[19] Crnko S , Du Pré BC , Sluijter JPG , Van Laake LW . Circadian rhythms and the molecular clock in cardiovascular biology and disease. Nat Rev Cardiol 2019 10.1038/s41569-019-0167-4 30796369

[20] Sato S , Sassone‐Corsi P . Circadian and epigenetic control of depression‐like behaviors. Curr Opin Behav Sci 2019; 25: 15–22.

[21] Jha PK , Challet E , Kalsbeek A . Circadian rhythms in glucose and lipid metabolism in nocturnal and diurnal mammals. Mol Cell Endocrinol 2015; 418: 74–88. 25662277 10.1016/j.mce.2015.01.024

[22] Bilu C *et al* Diurnality, type 2 diabetes, and depressive‐like behavior. J Biol Rhythms 2018; 34.1: 69–83. 10.1177/0748730418819373 30585103

[23] Staels B . When the Clock stops ticking, metabolic syndrome explodes. Nat Med 2006; 12: 54. 16397568 10.1038/nm0106-54

[24] Vgontzas AN , Bixier EO , Chrousos GP . Sleep apnea is a manifestation of the metabolic syndrome. Sleep Med Rev 2005; 9: 211–24. 15893251 10.1016/j.smrv.2005.01.006

[25] Gramaglia C *et al* Increased risk of metabolic syndrome in antidepressants users: a mini review. Front Psychiatry 2018; 9 10.3389/fpsyt.2018.00621 PMC6279880 30546325

[26] Javeed N , Matveyenko AV . Circadian etiology of type 2 diabetes mellitus. Physiology 2018; 33: 138–50. 29412061 10.1152/physiol.00003.2018 PMC5899235

[27] Yaffe K . Metabolic syndrome and cognitive disorders: is the sum greater than its parts? Alzheimer Dis Assoc Disord 2007; 21: 167–71. 17545744 10.1097/WAD.0b013e318065bfd6

[28] McIntyre RS *et al* Should depressive syndromes be reclassified as “metabolic syndrome type II”? Ann Clin Psychiatry 2007; 19: 257–64. 18058283 10.1080/10401230701653377

[29] Reinke H , Asher G . Circadian clock control of liver metabolic functions. Gastroenterology 2016; 150: 574–80. 26657326 10.1053/j.gastro.2015.11.043

[30] Smolensky MH , Hermida RC , Castriotta RJ , Portaluppi F . Role of sleep‐wake cycle on blood pressure circadian rhythms and hypertension. Sleep Med 2007; 8: 668–80. 17383936 10.1016/j.sleep.2006.11.011

[31] Skene DJ *et al* Separation of circadian‐and behavior‐driven metabolite rhythms in humans provides a window on peripheral oscillators and metabolism. Proc Natl Acad Sci 2018; 115: 7825–30. 29991600 10.1073/pnas.1801183115 PMC6065025

[32] Dibner C , Schibler U . Circadian timing of metabolism in animal models and humans. J Intern Med 2015; 277: 513–27. 25599827 10.1111/joim.12347

[33] Gale JE *et al* Disruption of circadian rhythms accelerates development of diabetes through pancreatic beta‐cell loss and dysfunction. J Biol Rhythms 2011; 26: 423–33. 21921296 10.1177/0748730411416341 PMC3359760

[34] Kadono M *et al* Various patterns of disrupted daily rest‐activity rhythmicity associated with diabetes. J Sleep Res 2016; 25: 426–37. 26853999 10.1111/jsr.12385

[35] Rakshit K , Thomas AP , Matveyenko AV . Does disruption of circadian rhythms contribute to beta‐cell failure in type 2 diabetes? Curr Diab Rep 2014; 14: 8. 10.1007/s11892-014-0474-4 PMC3988110 24532160

[36] Wyse CA *et al* Adverse metabolic and mental health outcomes associated with shiftwork in a population‐based study of 277,168 workers in UK biobank. Ann Med 2017; 49: 411–20. 28166415 10.1080/07853890.2017.1292045

[37] Spiegel K , Knutson K , Leproult R , Tasali E , Van Cauter E . Sleep loss: a novel risk factor for insulin resistance and type 2 diabetes. J Appl Physiol 2005; 99: 2008–19. 16227462 10.1152/japplphysiol.00660.2005

[38] Reaven GM . Role of insulin resistance in human disease. Diabetes 1988; 37: 1595–607. 3056758 10.2337/diab.37.12.1595

[39] Alberti KGM , Zimmet P , Shaw J . The metabolic syndrome—a new worldwide definition. Lancet 2005; 366: 1059–62. 16182882 10.1016/S0140-6736(05)67402-8

[40] Grundy SM . Does the metabolic syndrome exist?. Diabetes Care 2006; 29: 1689–92. 16801603 10.2337/dc05-2307

[41] Grant PJ . The clock stopped, never to go again … Diabetes and Vascular Disease Research 2008; 5: 75. 18537092 10.3132/dvdr.2008.0012

[42] Prasai MJ , George JT , Scott EM . Molecular clocks, type 2 diabetes and cardiovascular disease. Diab Vasc Dis Res 2008; 5: 89–95. 18537095 10.3132/dvdr.2008.015

[43] Reaven GM . The metabolic syndrome: time to get off the merry‐go‐round? J Intern Med 2011; 269: 127–36. 21129047 10.1111/j.1365-2796.2010.02325.x

[44] Alberti KGMM , Zimmet P , Shaw J . Metabolic syndrome—a new world‐wide definition. A consensus statement from the international diabetes federation. Diabet Med 2006; 23: 469–80. 16681555 10.1111/j.1464-5491.2006.01858.x

[45] Alberti K *et al* Harmonizing the metabolic syndrome: a joint interim statement of the international diabetes federation task force on epidemiology and prevention; national heart, lung, and blood institute; American heart association; world heart federation; international atherosclerosis society; and international association for the study of obesity. Circulation 2009; 120: 1640–5. 19805654 10.1161/CIRCULATIONAHA.109.192644

[46] Gluckman PD , Hanson MA , Buklijas T , Low FM , Beedle AS . Epigenetic mechanisms that underpin metabolic and cardiovascular diseases. Nat Rev Endocrinol 2009; 5: 401. 19488075 10.1038/nrendo.2009.102

[47] Barres R , Zierath JR . DNA methylation in metabolic disorders. Am J Clin Nutr 2011; 93: 897S–900S. 21289222 10.3945/ajcn.110.001933

[48] Keating ST , Plutzky J , El‐Osta A . Epigenetic changes in diabetes and cardiovascular risk. Circ Res 2016; 118: 1706–22. 27230637 10.1161/CIRCRESAHA.116.306819 PMC5593074

[49] Block T , El‐Osta A . Epigenetic programming, early life nutrition and the risk of metabolic disease. Atherosclerosis 2017; 266: 31–40. 28950165 10.1016/j.atherosclerosis.2017.09.003

[50] Keating ST , van Diepen JA , Riksen NP , El‐Osta A . Epigenetics in diabetic nephropathy, immunity and metabolism. Diabetologia 2018; 61: 6–20. 29128937 10.1007/s00125-017-4490-1 PMC6448927

[51] Kaspi A *et al* Diet during pregnancy is implicated in the regulation of hypothalamic RNA methylation and risk of obesity in offspring. Mol Nutr Food Res 2018; 62: e1800134 10.1002/mnfr.201800134 29882289

[52] Khurana I *et al* DNA methylation regulates hypothalamic gene expression linking parental diet during pregnancy to the offspring's risk of obesity in *Psammomys obesus* . Int J Obes (Lond) 2016; 40: 1079–88. 27108813 10.1038/ijo.2016.64

[53] Kahn R . The metabolic syndrome (emperor) wears no clothes. Diabetes Care 2006; 29: 1693–6. 10.2337/dc06-1453 17065717

[54] Kahn R , Buse J , Ferrannini E , Stern M . The metabolic syndrome: time for a critical appraisal. Diabetologia 2005; 48: 1684–99. 16079964 10.1007/s00125-005-1876-2

[55] Zimmet PZ , Alberti KGM . Epidemiology of diabetes—status of a pandemic and issues around metabolic surgery. Diabetes Care 2016; 39: 878–83. 27222545 10.2337/dc16-0273

[56] Cedernaes J *et al* Acute sleep loss results in tissue‐specific alterations in genome‐wide DNA methylation state and metabolic fuel utilization in humans. Sci Adv 2018; 4 10.1126/sciadv.aar8590 PMC6105229 30140739

[57] Van Laake LW , Luscher TF , Young ME . The circadian clock in cardiovascular regulation and disease: lessons from the nobel prize in physiology or medicine 2017. Eur Heart J 2018; 39: 2326–9. 29309706 10.1093/eurheartj/ehx775 PMC6012474

[58] Thosar SS , Butler MP , Shea SA . Role of the circadian system in cardiovascular disease. J Clin Invest 2018; 128: 2157–67. 29856365 10.1172/JCI80590 PMC5983320

[59] Shaw E , Tofler GH . Circadian rhythm and cardiovascular disease. Curr Atheroscler Rep 2009; 11: 289–95. 19500492 10.1007/s11883-009-0044-4

[60] Black N *et al* Circadian rhythm of cardiac electrophysiology, arrhythmogenesis, and the underlying mechanisms. Heart Rhythm 2018; 16: 298–307. 10.1016/j.hrthm.2018.08.026 30170229 PMC6520649

[61] Durgan DJ , Young ME . The cardiomyocyte circadian clock emerging roles in health and disease. Circ Res 2010; 106: 647–58. 20203314 10.1161/CIRCRESAHA.109.209957 PMC3223121

[62] Fabbian F *et al* Dipper and non‐dipper blood pressure 24‐hour patterns: circadian rhythm–dependent physiologic and pathophysiologic mechanisms. Chronobiol Int 2013; 30: 17–30. 23002916 10.3109/07420528.2012.715872

[63] Banegas JR *et al* Relationship between clinic and ambulatory blood‐pressure measurements and mortality. N Engl J Med 2018; 378: 1509–20. 29669232 10.1056/NEJMoa1712231

[64] Hassan MO *et al* Non‐dipping blood pressure in the metabolic syndrome among arabs of the oman family study. Obesity 2007; 15: 2445–53. 17925470 10.1038/oby.2007.290

[65] Schein AS , Kerkhoff AC , Coronel CC , Plentz RD , Sbruzzi G . Continuous positive airway pressure reduces blood pressure in patients with obstructive sleep apnea; a systematic review and meta‐analysis with 1000 patients. J Hypertens 2014; 32: 1762–73. 24979300 10.1097/HJH.0000000000000250

[66] Casitas R *et al* The effect of treatment for sleep apnoea on determinants of blood pressure control. Eur Respir J 2017; 50: 1701261. 29146604 10.1183/13993003.01261-2017

[67] Hermida RC *et al* Circadian rhythms in blood pressure regulation and optimization of hytertension treatment with ACE inhibitor and ARB medications. Am J Hypertens 2011; 24: 383–91. 20930708 10.1038/ajh.2010.217

[68] de la Sierra A *et al* Prevalence and factors associated with circadian blood pressure patterns in hypertensive patients. Hypertension 2009; 53: 466–72. 19171788 10.1161/HYPERTENSIONAHA.108.124008

[69] Adamovich Y *et al* Circadian clocks and feeding time regulate the oscillations and levels of hepatic triglycerides. Cell Metab 2014; 19: 319–30. 24506873 10.1016/j.cmet.2013.12.016 PMC4261230

[70] Gnocchi D , Pedrelli M , Hurt‐Camejo E , Parini P . Lipids around the clock: focus on circadian rhythms and lipid metabolism. Biology 2015; 4: 104. 25665169 10.3390/biology4010104 PMC4381220

[71] Bray MS , Young ME . Circadian rhythms in the development of obesity: potential role for the circadian clock within the adipocyte. Obes Rev 2007; 8: 169–81. 17300281 10.1111/j.1467-789X.2006.00277.x

[72] Froy O . Metabolism and circadian rhythms—implications for obesity. Endocr Rev 2010; 31: 1–24. 19854863 10.1210/er.2009-0014

[73] Stenvers DJ *et al* Diurnal rhythms in the white adipose tissue transcriptome are disturbed in obese individuals with type 2 diabetes compared with lean control individuals. Diabetologia 2019; 62: 704–16. 10.1007/s00125-019-4813-5 30737520

[74] Zimmet PZ , Wall JR , Rome R , Stimmler L , Jarrett RJ . Diurnal variation in glucose tolerance: associated changes in plasma insulin, growth hormone, and non‐esterified. BMJ 1974; 1: 485–8. 4817159 10.1136/bmj.1.5906.485 PMC1633483

[75] Panda S . Circadian physiology of metabolism. Science 2016; 354: 1008–15. 27885007 10.1126/science.aah4967 PMC7261592

[76] Coomans CP *et al* The suprachiasmatic nucleus controls circadian energy metabolism and hepatic insulin sensitivity. Diabetes 2013; 62: 1102–8. 23274903 10.2337/db12-0507 PMC3609590

[77] Kalsbeek A , la Fleur S , Fliers E . Circadian control of glucose metabolism. Mol Metab 2014; 3: 372–83. 24944897 10.1016/j.molmet.2014.03.002 PMC4060304

[78] Yu JS , Marsh S , Hu JB , Feng WK , Wu CD . The pathogenesis of nonalcoholic fatty liver disease: interplay between diet, gut microbiota, and genetic background. Gastroenterol Res Pract 2016 10.1155/2016/2862173 PMC4876215 27247565

[79] Egede LE , Zheng D , Simpson K . Comorbid depression is associated with increased health care use and expenditures in individuals with diabetes. Diabetes Care 2002; 25: 464–70. 11874931 10.2337/diacare.25.3.464

[80] Anderson RJ , Freedland KE , Clouse RE , Lustman PJ . The prevalence of comorbid depression in adults with diabetes – a meta‐analysis. Diabetes Care 2001; 24: 1069–78. 11375373 10.2337/diacare.24.6.1069

[81] Campayo A , Gomez‐Biel CH , Lobo A . Diabetes and depression. Curr Psychiatry Rep 2011; 13: 26–30. 21052874 10.1007/s11920-010-0165-z

[82] Golden SH *et al* Examining a bidirectional association between depressive symptoms and diabetes. JAMA 2008; 299: 2751–9. 18560002 10.1001/jama.299.23.2751 PMC2648841

[83] Bilu C , Einat H , Kronfeld‐Schor N . Utilization of diurnal rodents in the research of depression. Drug Dev Res 2016; 77: 336–45. 27654112 10.1002/ddr.21346

[84] Kronfeld‐Schor N , Einat H . Circadian rhythms and depression: human psychopathology and animal models. Neuropharmacology 2012; 62: 101–14. 21871466 10.1016/j.neuropharm.2011.08.020

[85] Logan RW , McClung CA . Rhythms of life: circadian disruption and brain disorders across the lifespan. Nat Rev Neurosci 2019; 20: 49–65. 30459365 10.1038/s41583-018-0088-y PMC6338075

[86] McClung CA . Circadian genes, rhythms and the biology of mood disorders. Pharmacol Ther 2007; 114: 222–32. 17395264 10.1016/j.pharmthera.2007.02.003 PMC1925042

[87] McClung CA . How might circadian rhythms control mood? Let me count the ways. Biol Psychiat 2013; 74: 242–9. 23558300 10.1016/j.biopsych.2013.02.019 PMC3725187

[88] Brouwer A *et al* Light therapy for better mood and insulin sensitivity in patients with major depression and type 2 diabetes: a randomised, double‐blind, parallel‐arm trial. BMC Psychiatry 2015; 15 10.1186/s12888-015-0543-5 PMC4513382 26204994

[89] Dietzel M , Saletu B , Lesch OM , Sieghart W , Schjerve M . Light treatment in depressive illness. Polysomnographic, psychometric and neuroendocrinological findings. Eur Neurol 1986; 25: 93–103. 10.1159/000116089 3758132

[90] Even C , Schroder CM , Friedman S , Rouillon F . Efficacy of light therapy in nonseasonal depression: a systematic review. J Affect Disord 2008; 108: 11–23. 17950467 10.1016/j.jad.2007.09.008

[91] Golden RN *et al* The efficacy of light therapy in the treatment of mood disorders: a review and meta‐analysis of the evidence. Am J Psychiatry 2005; 162: 656–62. 15800134 10.1176/appi.ajp.162.4.656

[92] Kripke DF . Light treatment for nonseasonal depression: speed, efficacy, and combined treatment. J Affect Disord 1998; 49: 109–17. 9609674 10.1016/s0165-0327(98)00005-6

[93] Oldham MA , Ciraulo DA . Bright light therapy for depression: a review of its effects on chronobiology and the autonomic nervous system. Chronobiol Int 2014; 31: 305–19. 24397276 10.3109/07420528.2013.833935 PMC5403163

[94] Terman M , Terman JS . Light therapy for seasonal and nonseasonal depression: efficacy, protocol, safety, and side effects. CNS Spectr 2005; 10: 647–63; quiz 672. 16041296 10.1017/s1092852900019611

[95] Wirz‐Justice A . Beginning to see the light. Arch Gen Psychiatry 1998; 55: 861–2. 9783554 10.1001/archpsyc.55.10.861

[96] Yamada N , Martin‐Iverson MT , Daimon K , Tsujimoto T , Takahashi S . Clinical and chronobiological effects of light therapy on nonseasonal affective disorders. Biol Psychiatry 1995; 37: 866–73. 7548461 10.1016/0006-3223(94)00221-N

[97] Almoosawi S *et al* Chronotype: implications for epidemiologic studies on chrono‐nutrition and cardiometabolic health. Adv Nutr 2018; 10: 30–42. 10.1093/advances/nmy070 PMC6370261 30500869

[98] El‐Osta A *et al* Transient high glucose causes persistent epigenetic changes and altered gene expression during subsequent normoglycemia. J Exp Med 2008; 205: 2409–17. 18809715 10.1084/jem.20081188 PMC2556800

[99] Okabe J *et al* Distinguishing hyperglycemic changes by Set7 in vascular endothelial cells. Circ Res 2012; 110: 1067–76. 22403242 10.1161/CIRCRESAHA.112.266171

[100] Barnett M , Collier GR , Zimmet P , Odea K . The effect of restricting energy‐intake on diabetes in *Psammomys‐obesus* . Int J Obes Relat Metab Disord 1994; 18: 789–94. 7894516

[101] Schmidt‐Nielsen K , Haines HB , Hackel DB . Diabetes mellitus in the sand rat induced by standard laboratory diets. Science 1964; 143: 689–90. 14081240 10.1126/science.143.3607.689

[102] Kalman R , Ziv E , Lazarovici G , Shafrir E . The Laboratory Rabbit, Guinea Pig, Hamster, and Other Rodents. London: Academic Press, 2012; 1171–90.

[103] Kaiser N , Cerasi E , Leibowitz G . Diet‐induced diabetes in the sand rat (*Psammomys obesus*)In Animal Models in Diabetes Research. Totowa, NJ: Humana Press, 2012; (pp. 89–102). 10.1007/978-1-62703-068-7_7 22893403

[104] Kronfeld‐Schor N , Visser ME , Salis L , van Gils JA . Chronobiology of interspecific interactions in a changing world. Philos Trans R Soc Lond B Biol Sci 2017; 372 10.1098/rstb.2016.0248 PMC5647275 28993492

[105] Einat H , Kronfeld‐Schor N , Eilam D . Sand rats see the light: short photoperiod induces a depression‐like response in a diurnal rodent. Behav Brain Res 2006; 173: 153–7. 16831474 10.1016/j.bbr.2006.06.006

[106] Ashkenazy T , Einat H , Kronfeld‐Schor N . Effects of bright light treatment on depression‐ and anxiety‐like behaviors of diurnal rodents maintained on a short daylight schedule. Behav Brain Res 2009; 201: 343–6. 19428655 10.1016/j.bbr.2009.03.005

[107] Ashkenazy T , Einat H , Kronfeld‐Schor N . We are in the dark here: induction of depression‐ and anxiety‐like behaviours in the diurnal fat sand rat, by short daylight or melatonin injections. Int J Neuropsychopharmacol 2009; 12: 83–93. 18631427 10.1017/S1461145708009115

[108] Krivisky K , Einat H , Kronfeld‐Schor N . Effects of morning compared with evening bright light administration to ameliorate short‐photoperiod induced depression‐ and anxiety‐like behaviors in a diurnal rodent model. J Neural Transm 2012; 119: 1241–8. 22407379 10.1007/s00702-012-0783-1

[109] Ashkenazy‐Frolinger T , Einat H , Kronfeld‐Schor N . Diurnal rodents as an advantageous model for affective disorders: novel data from diurnal degu (*Octodon degus*). J Neural Transm 2013; 35–45. 10.1007/s00702-013-1137-3 24352409

[110] Tal‐Krivisky K , Kronfeld‐Schor N , Einat H . Voluntary exercise enhances activity rhythms and ameliorates anxiety‐and depression‐like behaviors in the sand rat model of circadian rhythm‐related mood changes. Physiol Behav 2015; 151: 441–7. 26253214 10.1016/j.physbeh.2015.08.002

